# ﻿Two new species of the mealybug genus *Paracoccus* from Jiangxi, South China (Hemiptera, Coccomorpha, Pseudococcidae)

**DOI:** 10.3897/zookeys.1197.118778

**Published:** 2024-04-03

**Authors:** Jiang-Tao Zhang, Chao-Qun Li, Xing-Ping Liu, Yan Wang

**Affiliations:** 1 Key Laboratory of National Forestry and Grassland Administration on Forest Ecosystem Protection and Restoration of Poyang Lake Watershed, College of Forestry, Jiangxi Agricultural University, Nanchang, 330045, China Jiangxi Agricultural University Nanchang China; 2 College of Forestry, Jiangxi Agricultural University, Nanchang, 330045, China Jiangxi Agricultural University Nanchang China

**Keywords:** *Dalbergiahupeana*, key, *Pinusmassoniana*, Sternorrhyncha, taxonomy

## Abstract

Two new mealybug species, *Paracoccusgillianwatsonae* Zhang, **sp. nov.** and *P.wui* Zhang, **sp. nov.**, collected from Jiangxi, South China, are described and illustrated based on the morphology of adult females. *Paracoccusgillianwatsonae* is similar to *P.burnerae* (Brain, 1915), but it differs in having fewer pairs of cerarii, and in lacking both ventral oral collar tubular ducts on the margins of the head and translucent pores on the hind femur. *Paracoccuswui* resembles *P.keralae* Williams, 2004 and *P.neocarens* (Lit, 1992), but it differs in lacking ventral oral collar tubular ducts on the margins of the head and in having multilocular disc-pores usually in double rows at the posterior edges of abdominal segments V and VI. A key to the *Paracoccus* species found in China is provided.

## ﻿Introduction

Members of the family Pseudococcidae (commonly known as mealybugs) are small sap-sucking insects. The Pseudococcidae is the second-largest family of scale insects, with about 2047 described species in 261 genera worldwide (García Morales et al. 2016). The mealybug genus *Paracoccus* was established by Ezzat and McConnell (1956) with *Pseudococcusburnerae* Brain, 1915 designated as the type species. Currently, the genus includes 94 species (García Morales et al. 2016) and has a wide distribution in temperate and tropical parts of the world (Moghaddam and Esfandiari 2014). However, molecular phylogenetic analyses by Hardy et al. (2008) and Choi and Lee (2022) have indicated that *Paracoccus* is not monophyletic. Further molecular and morphological studies on this genus are greatly needed.

In China, Chen et al. (2011) first reported the invasive *Paracoccusmarginatus* Williams & Granara de Willink, 1992 from Taiwan; later, this species was found in many places in mainland China, e.g. Fujian (Lin et al. 2019), Guangdong (Ahmed et al. 2015), Hainan (Wang et al. 2018), Jiangxi (Liao et al. 2021), and Yunnan (Wu et al. 2014; Zhang and Wu 2015). In addition, Miller et al. (2014) reported that three *Paracoccus* species originating from China had been intercepted at U.S. ports of entry between 1995 and 2012: *P.burnerae*, *P.interceptus* Lit, 1997, and *P.marginatus*. Together with the two new species described herein, there are now five *Paracoccus* species reported from China.

In this study, *Paracoccusgillianwatsonae* and *P.wui*, both collected from Jiangxi, South China, are described and illustrated based on the morphology of adult females. A key to the species of *Paracoccus* reported from China is provided.

## ﻿Materials and methods

Adult female specimens were slide-mounted using the method described by Borchsenius (1950). The morphological terminology used follows that of Williams (2004). Body measurements are given in millimeters (mm) and measurements of other structures are given in micrometers (µm). Each taxonomic illustration of the adult female is arranged in the usual way for Coccomorpha, with features of the dorsum shown on the left side and those of the venter on the right. Enlargements of some important characters, not drawn to scale, are arranged around the main illustration. The letters and abbreviations used on the illustrations are explained in the legend below each illustration.

Slides of the new species are deposited in the College of Forestry, Jiangxi Agricultural University, Nanchang, China (**CFJAU**) and the Insect Collection of the Southwest Forestry University, Yunnan, China (**SWFU**). The slide labels are written in Chinese. Under ‘Materials examined’, the collection data of each holotype is listed with ‘/’ indicating the positions of the line breaks on the labels.

## ﻿Taxonomy

### ﻿Genus Paracoccus

Taxon classificationAnimaliaHemipteraPseudococcidae

Ezzat & McConnell, 1956

A0DF9F87-8B74-57B5-AEBF-637F6AB330E9

Paracoccus Ezzat and McConnell 1956: 37. Type species: Pseudococcusburnerae Brain, by original designation.
Gossypina Salazar 1972: 293. Type species: Gossypinaglauca Salazar, by monotypy and original designation. Synonymized by Williams and Granara de Willink 1992: 292.

#### Diagnosis.

(adapted and slightly modified from Williams 2004): body of adult female usually broadly oval. Anal lobes usually developed, each with a partial or complete ventral anal lobe bar extending from either apical seta or from bar seta. Antennae each normally 8-segmented. Legs well developed, claw without a denticle; tarsal digitules knobbed; translucent pores usually present on hind coxa and tibia. Cerarii numbering as many as 18 pairs, each cerarius bearing 2 conical setae, except some on head and thorax each with 3 or 4 setae; auxiliary setae usually absent except in anal lobe cerarii. Circulus present or absent. Anterior and posterior ostioles present. Anal ring normally with 6 setae. Oral rim tubular ducts present on dorsum and sometimes on venter. Oral collar tubular ducts present. Multilocular disc-pores present, at least on venter. Trilocular pores present.

### ﻿ Paracoccus gillianwatsonae

Taxon classificationAnimaliaHemipteraPseudococcidae

Zhang sp. nov.

799FA47B-8A01-5A38-8F96-74053553D4D6

https://zoobank.org/E9FB2EE0-028A-4C6A-96B0-D23596F5EC01

Figs 1 , 2

#### Materials examined.

***Holotype***: 1 ♀ (mounted singly on a slide), China, Jiangxi, Yichun City, Fengxin County, Chi’an Town [28°39′55″N, 115°21′9″E] / on the needles of *Pinusmassoniana* Lamb. (Pinaceae) / 22.ix.2022, coll. Jiang-tao Zhang (CFJAU). ***Paratypes***: 4 ♀♀ (mounted singly on 4 slides), same data as holotype (3 CFJAU, 1 SWFU).

#### Etymology.

The species is named after Dr Gillian W. Watson (Department of Life Sciences, the Natural History Museum, London, U.K.), who has selflessly helped the first author.

#### Description.

**Figure 1.**
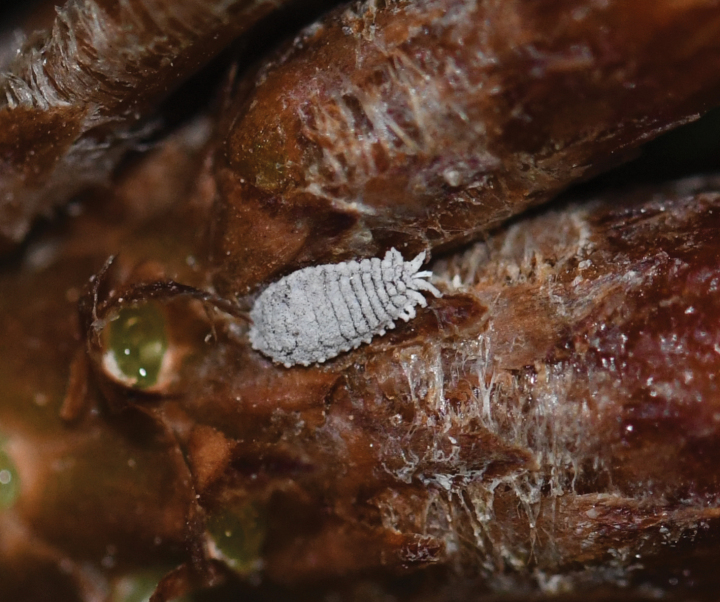
Live adult female of *Paracoccusgillianwatsonae* Zhang, sp. nov. on a needle of *Pinusmassoniana* Lamb. (Pinaceae).

**Live adult female** (Fig. 1). Body elongate-oval, covered with thin, pale grey, mealy wax and with short, white lateral filaments around posterior body margins; these becoming successively longer on posteriormost four segments, with caudal filaments longest, each about 1/3 as long as maximum body width.

**Figure 2.**
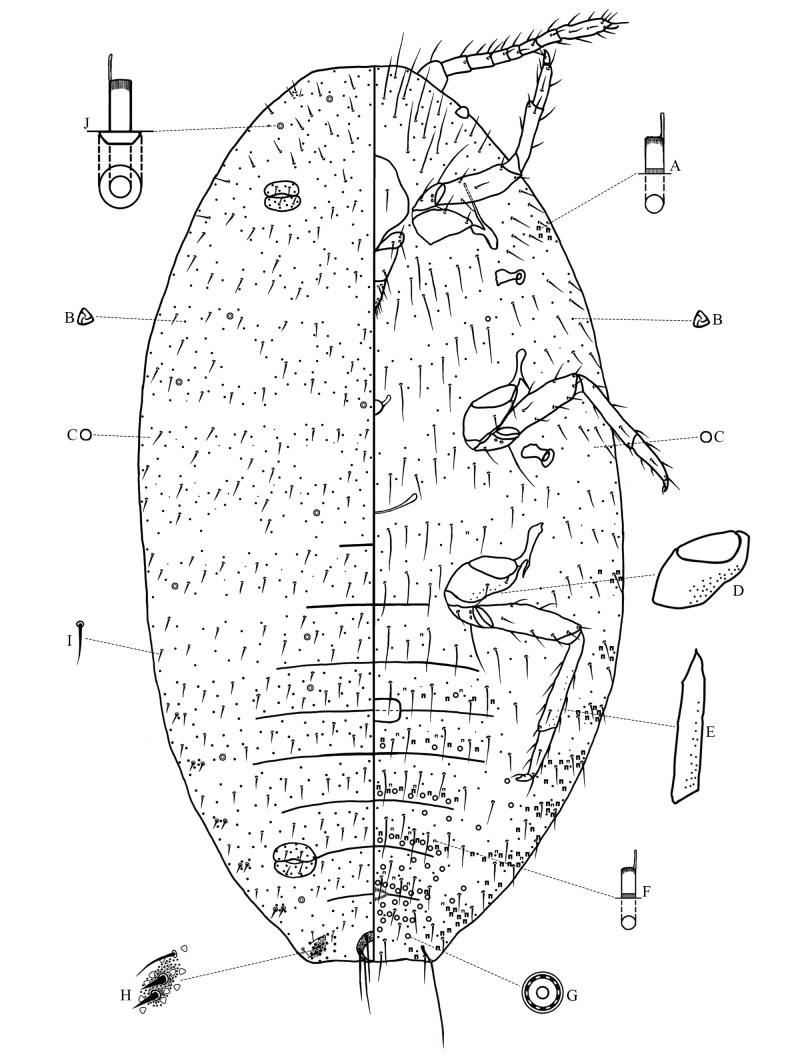
Adult female of *Paracoccusgillianwatsonae* Zhang, sp. nov. **A** large-type oral collar tubular duct **B** trilocular pore **C** discoidal pore **D** hind coxa **E** hind tibia **F** small-type oral collar tubular duct **G** multilocular disc-pore **H** anal lobe cerarius (C_18_) **I** dorsal seta **J** oral rim tubular duct.

**Slide-mounted adult female** (Fig. 2, *n* = 5). Body elongate-oval, 1.4–1.8 mm long and 0.8–1.0 mm wide. Anal lobes moderately developed, dorsal surface of each lobe with a small, slightly sclerotized area; ventral surface with long apical seta, each 180–200 µm long, and anal lobe bar present forwards from apical seta. Antennae each usually with 8 segments, sometimes with segments IV and V combined; each antenna 322–360 µm long, length of segments (in µm): I 35.0–37.5, II 42.5–47.5, III 42.5–50.0, IV 25.0–30.0, V 22.5–30.0, VI 30.0–37.5, VII 35.0–40.0 and VIII 85.0–95.0. Eye spot located on body margin lateral to antennal base. Clypeolabral shield 150–163 µm long and 120–140 µm wide. Labium 130–148 µm long and 80–95 µm wide, 0.87–0.98 times as long as clypeolabral shield. Legs well developed; claw stout, without a denticle; both tarsal digitules and claw digitules apically knobbed, longer than claw; with translucent pores present on hind coxa and tibia (Fig. 2D, E). Hind leg measurements (in µm): coxa 55–65, trochanter + femur 227–245, tibia + tarsus 242–278, claw 20.0–22.5. Ratio of lengths of hind tibia + tarsus to trochanter + femur 1: 1.10–1.19, ratio of lengths of hind tibia to tarsus 1: 1.67–1.85. Circulus nearly quadrate, 50–75 µm long and 65–85 µm wide, situated between abdominal segments III and IV, divided by an intersegmental line. Anterior and posterior ostioles present, each lip with 7–12 trilocular pores and 0–4 setae. Anal ring 65–80 µm wide, bearing 6 long setae, each seta 112–130 µm long. Cerarii usually numbering 6 or 7 pairs on abdomen, sometimes also with 1 pair present on thorax and 1 pair present on head. Anal lobe cerarii (C_18_) (Fig. 2H) each bearing 2 conical setae, each seta 15–17 µm long and 6–7 µm wide at base, with 1 or 2 auxiliary setae and 10–12 trilocular pores, all situated on a small slightly sclerotized area. Other cerarii each containing 2 conical setae and 3–5 trilocular pores. Cerarii C_12_, if present, usually each containing only 1 conical seta and 1 short seta. Cerarii on thorax, if present, usually each containing 2 slender conical setae. On head, usually the ocular cerarii (C_3_) present, each containing 3 or 4 slender setae. Discoidal pores (Fig. 2C), each narrower than a trilocular pore, scattered on dorsum and venter.

***Dorsum*.** Setae (Fig. 2I) short, stiff, sometimes spine-like, mostly each 20.0–32.5 μm long, accompanied by shorter setae, each 7.5–15.0 µm long; setal bases not closely associated with trilocular pores. Trilocular pores (Fig. 2B), each 3.0–3.5 µm wide, evenly distributed. Oral rim tubular ducts (Fig. 2J) present, numbering 11–24 in total, each duct 11–12 µm long and about 4 µm wide, with rim 8–9 µm in diameter, usually present singly near margins of head, thorax, and close to cerarii on posterior abdominal segments, and others usually present in medial or submedial areas of thorax and abdominal segments II and III, or only on abdominal segment II.

***Venter.*** Setae relatively long and flagellate, longest setae present on medial area of head and posterior abdominal segments, each 62.5–125.0 µm long, accompanied by shorter setae, each 20.0–32.5 µm long. Cisanal setae each 50–55 µm long; obanal setae each 45–50 µm long. Trilocular pores similar to those on dorsum, evenly distributed. Multilocular disc-pores (Fig. 2G), each 7–8 µm in diameter, present around vulva in more-or-less single rows at posterior edges of abdominal segments IV–VII, and scattered or in rows at anterior edges of abdominal segments VI–VII, sometimes with 2 pores on abdominal segment III; also sparsely present on margins of posterior abdominal segments, and occasionally in submedial areas of thorax. Oral collar tubular ducts of 2 main sizes present: larger type (Fig. 2A), each 6–7 µm long and about 3.5 µm wide, present on medial areas of abdominal segments III‒VII, in marginal groups on all abdominal segments and opposite each anterior coxa; and smaller type (Fig. 2F), each 5–6 µm long and 2.0–2.5 µm wide, present on medial areas of abdominal segments III‒VII, also sparsely present in marginal areas together with large ducts, and occasionally present in medial areas of thorax.

#### Host plant.

Pinaceae: *Pinusmassoniana* Lamb.

#### Distribution.

China (Jiangxi).

#### Remarks.

*Paracoccusgillianwatsonae* sp. nov. is similar to *P.burnerae* (Brain, 1915) (morphological characteristics of *P.burnerae* based on the redescription and illustration by Williams (2004)) in having ventral multilocular disc-pores in single rows at the posterior edges of abdominal segments IV and V; it differs from the latter (character states of *P.burnerae* given in parentheses) by having: (i) ventral oral collar tubular ducts absent from margins of the head (present); (ii) cerarii numbering fewer than 18 pairs (18 pairs); and (iii) translucent pores absent from hind femur (present).

### ﻿ Paracoccus wui

Taxon classificationAnimaliaHemipteraPseudococcidae

Zhang sp. nov.

459EDF41-7CDA-5CCE-9CE5-C82FCFE86024

https://zoobank.org/150B72B5-4F10-4795-9FC4-C67A338FE302

Figs 3 , 4

#### Materials examined.

***Holotype***: 1 ♀ (mounted singly on a slide), China, Jiangxi, Lushan National Nature Reserve [29°33′N, 115°57′E] / under bark crack of *Dalbergiahupeana* Hance (Fabaceae) / 14.ix.2021, coll. Jiang-tao Zhang (CFJAU). ***Paratypes***: 8 ♀♀ (mounted singly on 8 slides), same data as holotype (6 CFJAU, 2 SWFU).

#### Etymology.

The species is named after Dr San-an Wu (Beijing Forestry University, Beijing, China), who has made important contributions to the study of Chinese scale insects and has selflessly helped the first author.

#### Description.

**Figure 3.**
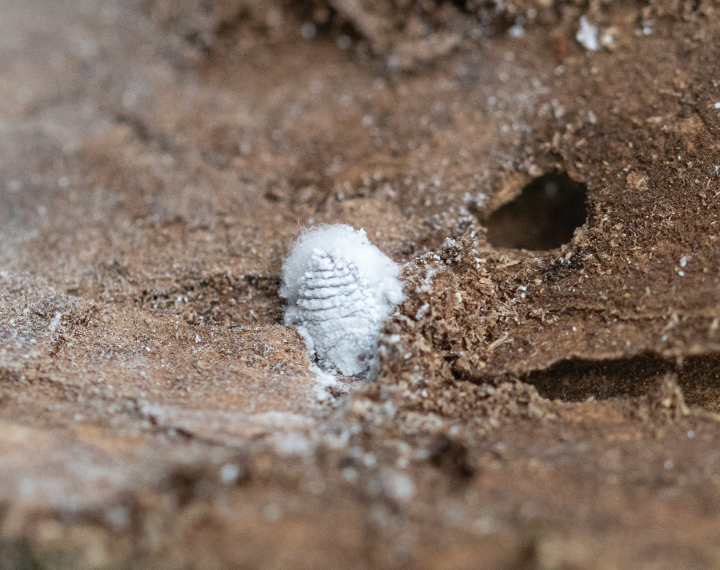
Live adult female of *Paracoccuswui* Zhang, sp. nov. in bark crack of *Dalbergiahupeana* Hance (Fabaceae).

**Live adult female** (Fig. 3). Body of mature adult female elongate-oval, covered with white, mealy wax; posterior of body with a short, white ovisac formed of tangled wax filaments.

**Figure 4.**
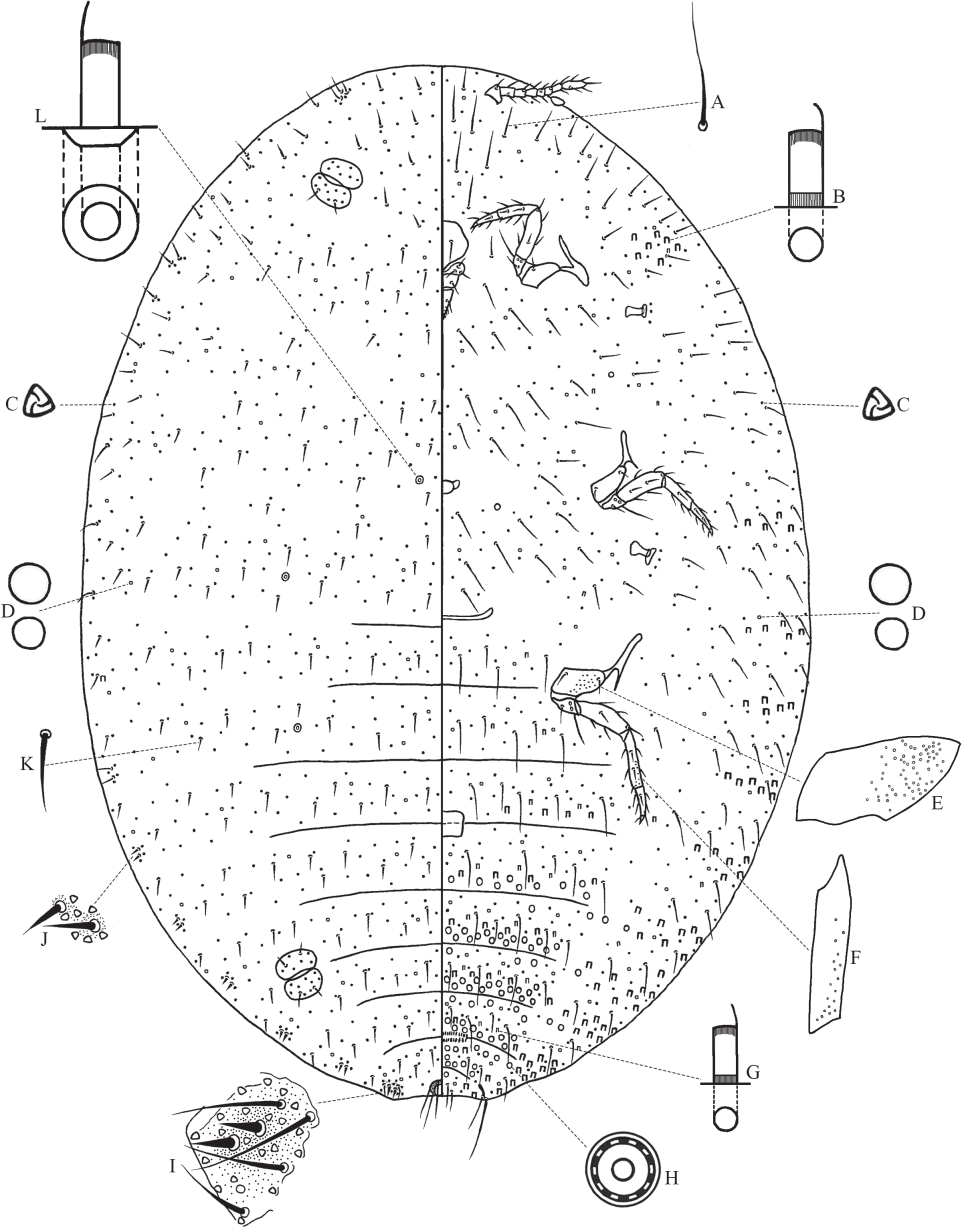
Adult female of *Paracoccuswui* Zhang, sp. nov. **A** ventral seta **B** large-type oral collar tubular duct **C** trilocular pore **D** discoidal pores **E** hind coxa **F** hind tibia **G** small-type oral collar tubular duct **H** multilocular disc-pore **I** anal lobe cerarius (C_18_) **J** cerarius on abdomen **K** dorsal seta **L** oral rim tubular duct.

**Slide-mounted adult female** (Fig. 4, *n* = 5). Body elongate-oval to broadly oval, 2.8–3.4 mm long and 1.9–2.5 mm wide. Anal lobes moderately developed, dorsal surface of each lobe with a small, slightly sclerotized area; ventral surface with long apical seta, each seta 225–240 µm long, and anal lobe bar present forwards from apical seta. Antennae each 355–458 µm long with 8 segments, length of segments (in µm): I 42.5–55.0, II 50.0–62.5, III 47.5–62.5, IV 25–35, V 27.5–50.0, VI 30.0–37.5, VII 37.5–50.0 and VIII 87.5–110.0. Eye spot located on body margin lateral to antennal base. Clypeolabral shield 163–213 µm long and 150–195 µm wide. Labium 178–225 µm long and 87.5–100.0 µm wide, 1.03–1.18 times as long as clypeolabral shield. Legs well developed; claw stout, without a denticle; both tarsal digitules and claw digitules apically knobbed, longer than claw; with translucent pores present on anterior and posterior surfaces of hind coxa (Fig. 4E) and on posterior surface of hind tibia (Fig. 4F). Hind leg measurements (in µm): coxa 80–113, trochanter + femur 275–378, tibia + tarsus 283–360, claw 27.5–32.5. Ratio of lengths of hind tibia + tarsus to trochanter + femur 1: 0.95–1.04, ratio of lengths of hind tibia to tarsus 1: 1.78–2.35. Circulus nearly quadrate, 115–145 µm long and 125–188 µm wide, situated between abdominal segments III and IV, divided by an intersegmental line. Anterior and posterior ostioles present, each lip with some trilocular pores and 1–5 setae. Anal ring 87.5–112.5 µm wide, bearing 6 long setae, each 120–145 µm long; occasionally 2 short setae present between long setae. Cerarii numbering 5–8 recognisable pairs on abdomen, sometimes also possibly 1–3 pairs discernible on thorax and head. Anal lobe cerarii (Fig. 4I) each containing 2 conical setae, each seta 16–20 µm long and 7–8 µm wide at base, 2–5 auxiliary setae and 22–26 trilocular pores, all situated on a small slightly sclerotized area. Anterior abdominal cerarii (Fig. 4J) each usually containing 2 smaller conical setae, 4–8 trilocular pores, and sometimes with small slightly sclerotized at setal bases; the setae in anterior abdominal cerarii sometimes widely spaced, or each reduced to only 1 conical seta, or replaced by 1 or 2 stout setae. Cerarii on thorax and head difficult to recognize because some marginal dorsal setae sometimes associated with 2–4 trilocular pores near setal bases, which can be confused with a cerarius; if cerarii present, each often with a pair of stout setae similar to those on dorsum, but recognisable as cerarius by a small concentration of trilocular pores, and sometimes the ocular cerarii (C_3_) present, each with 3 or 4 slender setae. Discoidal pores (Fig. 4D) of varying sizes, with larger pores each usually wider than a trilocular pore (in one paratype most larger pores about same diameter as a trilocular pore and only a few pores wider than a trilocular pore), scattered on dorsum and venter.

***Dorsum*.** Setae (Fig. 4K) stout with flagellate tips, sometimes bifurcate; larger setae, each 42.5–62.5 µm long and sometimes with 1 or 2 trilocular pores near setal base, and shorter setae, each 17.5–37.5 µm long. Trilocular pores (Fig. 4C), each 3–4 µm wide, evenly distributed. Multilocular disc-pores absent. Oral rim tubular ducts (Fig. 4L) very few, totaling 1–8 in available specimens, each about 9 µm long and 4–5 µm wide, with rim 8–9 µm in diameter, mainly present medially, submedially or submarginally on thorax and anterior abdominal segments, sometimes singly next to abdominal cerarii C_11_, C_15_ and C_17_, ducts usually absent from medially and submedially areas of abdominal segments IV–VIII. Oral collar tubular ducts usually absent from dorsum, occasionally 1 type of duct, similar to large type of oral collar tubular ducts on venter, singly present marginally or submarginally on thorax, or abdominal segment I, or abdominal segment IV.

***Venter*.** Setae (Fig. 4A) relatively long and flagellate, longest setae present on medial area of head and posterior abdominal segments, each 100–138 µm long, and shorter setae, each 30.0–62.5 µm long. Cisanal setae each 100–113 µm long; obanal setae each 95–105 µm long. Trilocular pores similar to those on dorsum, evenly distributed. Multilocular disc-pores (Fig. 4H), each 7–8 µm in diameter, present around vulva, usually in double rows at posterior edges of abdominal segments V–VII, and in single or double rows at the posterior edge of abdominal segment IV; others distributed at anterior edges of abdominal segments V–VII; in addition, a few pores located on submargins of abdominal segments and sometimes also present on abdominal segment III; also scattered pores present on medial and submedial areas of thorax. Oral collar tubular ducts of 2 main sizes present: larger type (Fig. 4B), each about 8 µm long and 3.5–4.0 µm wide, present mainly on medial areas of abdominal segments III‒VII, in marginal groups on thorax and abdomen, also opposite each anterior coxa; and smaller type (Fig. 4G), each about 6 µm long and 2.5–3.0 µm wide, present mainly on medial areas of abdominal segments II‒VII, also sparsely present in marginal areas together with large ducts, and occasionally in medial areas of thorax.

#### Host plant.

Fabaceae: *Dalbergiahupeana* Hance.

#### Distribution.

China (Jiangxi).

#### Remarks.

*Paracoccuswui* sp. nov. is similar to *P.keralae* Williams, 2004 in having only a few dorsal oral rim tubular ducts; it differs from the latter (character states of *P.keralae* given in parentheses) by having: (i) ventral oral collar tubular ducts absent from margins of the head (present); (ii) multilocular disc-pores usually in double rows at posterior edges of abdominal segments V and VI (in single rows); and (iii) larger discoidal pores usually each wider than a trilocular pore (narrower than a trilocular pore).

*Paracoccuswui* also resembles *P.neocarens* (Lit, 1992) (based on the redescription and illustration by Williams (2004)) in possessing some large discoidal pores and a reduced number of dorsal oral rim tubular ducts; it differs from the latter (character states of *P.neocarens* in parentheses) in having: (i) ventral oral collar tubular ducts absent from margins of the head (present between antennal bases); and (ii) multilocular disc-pores usually in double rows at posterior edges of abdominal segments V and VI (in single rows).

### ﻿Key to adult females of *Paracoccus* known from China

**Table:** 

1	Oral rim tubular ducts present on both dorsum and venter, near margins only	***P.marginatus* Williams & Granara de Willink**
–	Oral rim tubular ducts present on dorsum only, with at least a few in submedian or median areas	**2**
2	Ventral oral collar tubular ducts present on margins of the head	***P.burnerae* (Brain)**
–	Ventral oral collar tubular ducts absent from margins of the head	**3**
3	Abdominal segments IV and V with multilocular disc-pores present in single rows; labium shorter than clypeolabral shield	***P.gillianwatsonae* sp. nov.**
–	Abdominal segments IV and V with multilocular disc-pores present usually in double rows; labium longer than clypeolabral shield	**4**
4	Cerarii numbering 18 pairs; discoidal pores each narrower than a trilocular pore	***P.interceptus* Lit**
–	Cerarii numbering fewer than 18 pairs; larger discoidal pores each usually wider than a trilocular pore	***P.wui* sp. nov.**

## Supplementary Material

XML Treatment for Paracoccus

XML Treatment for Paracoccus gillianwatsonae

XML Treatment for Paracoccus wui

## ﻿Additional information

### Author ORCIDs

Jiang-tao Zhang https://orcid.org/0000-0002-0007-3918

Chao-qun Li https://orcid.org/0009-0004-5636-6906

Xing-ping Liu https://orcid.org/0000-0003-1831-9290

Yan Wang https://orcid.org/0009-0001-9347-6272

### Data availability

All of the data that support the findings of this study are available in the main text.
